# Impact of Co-Substrates on the Production of Poly(3-hydroxybutyrate-co-3-hydroxyvalerate) by *Burkholderia thailandensis* E264

**DOI:** 10.3390/ma18153577

**Published:** 2025-07-30

**Authors:** Jonathan Uriel Hernández-Alonso, María Alejandra Pichardo-Sánchez, Sergio Huerta-Ochoa, Angélica Román-Guerrero, Oliverio Rodríguez-Fernández, Humberto Vázquez-Torres, Roberto Olayo-González, Roberto Olayo-Valles, Luis Víctor Rodríguez-Durán, Lilia Arely Prado-Barragán

**Affiliations:** 1 Department of Biotechnology, Autonomous Metropolitan University Iztapalapa, Av. F.C. San Rafael Atlixco No. 186, Col. Leyes de Reforma 1 A Secc., Mexico City C.P. 09340, Mexico; uriel.alonso1998@gmail.com (J.U.H.-A.); alebiot@xanum.uam.mx (M.A.P.-S.); sho@xanum.uam.mx (S.H.-O.); arogue@xanum.uam.mx (A.R.-G.); 2 Polymer Processing, Center for Research in Applied Chemistry, Blvd. Enrique Reyna Hermosillo No. 140, Saltillo C.P. 25294, Coahuila, Mexico; oliverio.rodriguez@ciqa.edu.mx; 3 Department of Physics, Autonomous Metropolitan University Iztapalapa, Av. F.C. San Rafael Atlixco No. 186, Col. Leyes de Reforma 1 A Secc., Mexico City C.P. 09340, Mexico; hvto@xanum.uam.mx (H.V.-T.); oagr@xanum.uam.mx (R.O.-G.); rolv@xanum.uam.mx (R.O.-V.); 4 Mante Multidisciplinary Academic Unit, Autonomous University of Tamaulipas, E. Cárdenas González No. 1201 PTE., Col. Jardín, Ciudad Mante C.P. 89840, Tamaulipas, Mexico; luis.duran@docentes.uat.edu.mx

**Keywords:** *Burkholderia thailandensis* E264, levulinic acid, valeric acid, sodium propionate, biopolymers, PHB, PHBV

## Abstract

The synthesis of bioplastics from renewable resources is essential for green living. PHBV (poly(3-hydroxybutyrate-co-3-hydroxyvalerate)) is a biodegradable and biocompatible material ideal for various industrial applications. The impact of levulinic (LA), valeric acids (VA), and sodium propionate (SPr) as co-substrates in biomass and the synthesis of 3-hydroxy valerate (3HV) and co-polymerization of PHBV by *Burkholderia thailandensis* E264 (BtE264) was assessed. Thermogravimetric, XRD, NMR, and mechanical characterization were performed on the homopolymer (PHB) and co-polymer (PHBV), and compared to the PHBV-STD. BtE264 produced the co-polymer PHBV when adding any of the three co-substrates. LA showed a higher positive effect on microbial growth (8.4 g∙L^−1^) and PHBV production (3.91 g∙L^−1^), representing 78 and 22 mol % of 3HB and 3HV, respectively. The PHBV obtained with LA had a melting temperature (Tm) lower than the PHB homopolymer and presented lower values for melting enthalpies (ΔHf); the degree of crystallization and TGA values indicated that PHBV had better thermal stability. Additionally, FTIR and NMR revealed that BtE264 synthesizes PHBV with an organization in monomeric units (3HB-3HV), suggesting differentiated incorporation of the monomers, improving 3.4 times the break elongation the co-polymer’s tensile properties. This study highlights the co-substrates’ relevance in PHBV synthesis using BtE264 for the first time.

## 1. Introduction

Since the first synthetic plastic, called “Bakelite” (1907), plastic polymers have had a significant presence in everyday life, with their low production cost, lightness, and durability being the main factors that drove their production worldwide [1]. Currently, these virtues have triggered a problem in waste management, since most synthetic plastics have little or no biodegradability. Approximately 50% are discarded after a single use, causing them to end up in landfills, marine environments, soils, and other natural environments, fragmenting into microplastics and nanoplastics [2]. This makes them a potential threat, being able to alter the metabolic systems of plants (inhibiting or reducing their growth, hindering photosynthesis) or affecting the endocrine systems in animals when there is a risk of ingestion in the body. The degradation of a single fragment of microplastics can generate millions to billions of nano-sized particles. Despite their relatively small size, these fragments exhibit high stability and durability, with a potential permanence of hundreds to thousands of years, representing a danger to human health [3,4,5,6,7].

Therefore, sustainable alternatives to synthetic plastics are needed, with biopolymers being a promising option, as they have physical characteristics similar to synthetic plastics [8]. Currently, polyhydroxyalkanoates (PHA’s) have aroused wide interest in academia and industry, since they are a group of biodegradable, biocompatible, and biologically derived polyesters produced by microorganisms as a survival mechanism, being stored in the form of intracellular granules as a source of carbon and energy, when an essential nutrient is limited (nitrogen, phosphorus, or sulfur) in the presence of an excess carbon source [9,10]. The incorporation of the 3HV monomer into the 3HB chains results in a co-polymer with a less crystalline structure, lower melting temperature, lower Young’s modulus, greater flexibility, and break elongation, which are modified depending on the % mol of 3HV present in the chains [11,12]. Poly(3-hydroxybutyrate-co-3-hydroxyvalerate) (PHBV) is a bio-based polymer that is part of the PHA family composed of the union of two monomers, such as 3-hydroxybutyrate (3HB) and 3-hydroxyvalerate (3HV), which, unlike the homopolymer poly(3-hydroxybutyrate) (PHB), has better thermal and mechanical properties which give it a significant advantage in the development of biodegradable products. This polymer is biocompatible and non-toxic, and these characteristics are highly demanded in food packaging, controlled drug release, and tissue engineering, as well as in blends for 3D printing and electrospun nanofiber [13,14,15,16].

A key factor for the production of PHBV is the incorporation of precursors that serve in the synthesis of monomeric units of 3HV, because they are most responsible for the formation of the co-polymer when the microorganism has the metabolic capacity to synthesize them. Precursors are used as alternative or complementary carbon sources in combination with a main substrate, where sodium propionate (SPr), valeric acid (VA), levulinic acid (LA), and to a lesser extent propanol are some of the most studied precursors for the formation of the PHBV co-polymer [17,18]. In addition, many of the precursors can be obtained either through chemical synthesis or from organic sources such as SPr (wood pulp fermentation), VA (commonly extracted from the *Valeriana wallichii* plant), and LA (cellulosic biomass hydrolysis) [19,20,21]. Although the incorporation of precursors in the form of co-substrates favors the increase of the % mol of 3HV, high concentrations can inhibit cell growth, and therefore, maintaining control in the concentration of the co-substrate that is added to the culture medium or adjusting the concentrations along the kinetics are strategies used to increase the 3HV fraction without affecting the overall product yield [22,23].

In the present work, the production of PHBV by *Burkholderia thailandensis* E264 is demonstrated for the first time. The effect of different co-substrates (SPr, VA, and LA), and the thermal, mechanical, as well as molecular structure properties of the biopolymer obtained were characterized.

## 2. Materials and Methods

### 2.1. Bacterial Strain and Fermentation Conditions

*Burkholderia thailandensis* E264 (ATCC 700388) was prepared from a glycerol (20% *v*/*v*) to reseed nutrient agar plates. The inoculum was prepared by transferring 5 mL of the active strain to a 250 mL Erlenmeyer flask (EF) containing nutrient broth (NB) with a working volume of 50 mL and incubated 24 h at 30 °C and 150 rpm.

The experiments were carried out in a baffled EF with 250 mL, with a working volume of 50 mL. The culture medium reported by Aljuraifani et al. (2019) [24], with slight modifications, was used. The base production medium (BPM) was composed of (g·L^−1^): urea (2.50), KH_2_PO_4_ (1.50), Na_2_HPO_4_ (3.50), MgSO_4_∙7H_2_O (0.20), glycerol (20), and 1 mL trace element solution (1 mM FeSO_4_∙4H_2_O, CaCl_2_∙2H_2_O, MnSO_4_∙4H_2_O, ZnCl_2_). The addition of three co-substrates was evaluated, SPr (6 g·L^−1^), VA (4 g·L^−1^), and LA (4 g·L^−1^), adjusted to a similar carbon mol content in the three cultures and NaOH (2 N) was used to neutralize the acidic character of the co-substrates (pH 7–7.2) [25,26]. The culture media were tested individually, and each was assessed in triplicate. Additionally, a control BPM was prepared using glycerol as the sole carbon source (without co-substrate addition). Cultures were inoculated with 5% (*v*/*v*) and incubated for 168 h at 30 °C and 150 rpm. Samples (1.8 mL) were taken every 24 h, and microbial growth, substrate consumption, and PHBV production were determined.

### 2.2. Determination of Dry Biomass

Biomass was measured gravimetrically; cells were recovered by centrifugation; 1.8 mL of the microbial culture was placed in pre-weighed microtubes, then centrifuged (Eppendorf^®^, 5418, Hamburg, Germany) at 8000× *g* for 20 min, and the supernatant was transferred to a new microtube. The supernatant sample was kept frozen until later analysis of substrate consumption by high-performance liquid chromatography (HPLC). The cells were washed (2X) with distilled water, then dehydrated at 60 °C to a constant weight (12 h), allowed to cool in a desiccator, and weighed on an analytical balance. The biomass was determined by weight difference [24].

### 2.3. Analysis of Substrate Consumption

The initial and residual substrate and co-substrates (glycerol, LA, VA, and SPr) were analyzed by HPLC (Waters^®^ 2695, Milford, MA, USA) equipped with a refractive index detector (RID, Model 2414, Waters^®^ 2695, Milford, MA, USA), a photodiode array detector (PDA, Model 2996, Waters^®^ 2695, Milford, MA, USA), and a column furnace. Separation was performed in an IC-Pak ion-exclusion column (300 mm × 7.8 mm × 7 μm) using an isocratic method with H_2_SO_4_, 5 mM as the mobile phase at a flow of 0.6 mL∙min^−1^ at 37 °C using a 20 μL injection volume, following the method described by Ashby et al. (2018) [25] and Tao et al. (2011) [27] with slight modifications. Quantification was performed by the external standard method. Glycerol quantification was achieved with an RID detector, and co-substrate analysis with a PDA, SPr, and VA (210 nm) and LA (260 nm) detector. The samples were filtered (0.2 μm nylon) before analysis.

#### PHA Quantification

The determination of PHA was performed by gas chromatography (GC) according to the method reported by Juengert et al. (2018) [28] with slight modifications. Freeze-dried samples of 2–10 mg were weighed into test tubes, then 1 mL of chloroform with 1 mL of acidified methanol (15% *v*/*v* H_2_SO_4_) was added and incubated in a thermoreactor (Thermo Scientific^®^ Orion^®^ AQUAfast COD165, Waltham^®^, MA, USA) at 100 °C for 150 min.

The quantification and monomeric composition of the PHA produced was carried out by measuring the methyl esters of 3HB and 3HV on a gas chromatograph (Agilent-Technologies^®^ 7820A, Santa Clara, CA, USA) equipped with an autosampler (G-4513a Agilent-Technologies^®^, Santa Clara, CA, USA), a DB-Heavywax column (60 m × 0.250 mm × 0.25 μm) and a flame ionization detector. Two μL of sample was injected, using nitrogen as a carrier gas at a flow rate of 1.5 mL∙min^−1^. The initial oven temperature was set at 60 °C and then increased at a rate of 10 °C∙min^−1^ for 7 min and 30 °C∙min^−1^ until a maximum temperature of 250 °C was reached, and subsequently remained constant for 3 min. The injector and detector temperatures were 250 °C [29,30].

The total amount of PHBV was calculated by adding the detected amounts of 3HB and 3HV. The molar fraction of 3HV (% mol) was calculated as indicated in Equation (1):

(1) $$\%\mathrm{mol}3HV=\left(\frac{\mathrm{mol}3HV}{\mathrm{mol}3HB+\mathrm{mol}3HV}\right)\cdot 100$$

where the molar mass of 3HB = 104.1 g·mol^−1^ and for 3HV = 118.3 g·mol^−1^, which are the ratio between 3HV and the sum of 3HB and 3HV. A PHBV-STD (Sigma-Aldrich^®^, St. Louis, MO, USA) composed of 8% 3HV and 92% 3HB was used as an external standard [30].

### 2.4. PHA Characterization

#### 2.4.1. PHA Recovery

The PHA was recovered using the technique reported by Aljuraifani et al. (2019) [24] with slight modifications. The biomass obtained was dispersed in a commercial solution of sodium hypochlorite (Clorox^®^, Mexico City, Mexico ), added in a 1:1 (*v*/*v*) ratio relative to the initial culture volume, and incubated at 45 °C for 2 h, then centrifuged at 8000× *g* for 20 min. The PHA sediment was washed once with distilled water and twice with an ethanol–acetone (2:1) mixture. Afterwards, the sediment was dissolved in chloroform and centrifuged at 8000× *g* for 20 min. The recovered solid was poured into a Petri dish, and the solvent was evaporated for 12 h at room temperature.

#### 2.4.2. Thermal Analysis

The thermal properties of the polymer obtained were determined using a differential scanning calorimeter (DSC) and a thermogravimetric analyzer (TGA).

DSC analyses were performed using a TA Instruments^®^ Model Q 200 Thermal Analyzer (New Castle, DE, USA). The study was conducted on an aluminum pan with approximately 10 mg polymer, at a temperature range of −50 to 200 °C, with an increase of 10 °C∙min^−1^, and nitrogen flow of 50 mL∙min^−1^. A first heating was performed to obtain the endothermic melting peaks starting from −50 °C, up to 200 °C at 10 °C∙min^−1^ to eliminate the thermal history of the sample, then the samples were cooled from 200 to −50 °C. Then, a second run was made at a heating rate of 10 °C∙min^−1^ until 200 °C. The glass transition temperature (Tg), melting temperature (Tm), melting enthalpy (∆Hm), crystallization temperature (Tc), crystallization enthalpy (∆Hc), and % crystallization (% Xc) were determined from the thermogram of the second heating cycle [25]. The % Xc of the samples was determined by applying Equation (2):

(2) $$\%Xc=\left(\frac{∆H_{m}}{{∆H}_{\;\;\;mpolymer100\%}^{0}}\;\right)\cdot 100$$

where $\;∆H_{m}$ (J∙g^−1^) is the measured melting enthalpy and ${∆H}_{\;m}^{0}$ is the calculated melting enthalpy PHB (146 J∙g^−1^) and PHBV (109 J∙g^−1^) considering the 100% crystalline polymer [31,32].

TGA analysis was performed on a TA Instruments Model Q 500 (New Castle, DE, USA) to evaluate the thermal stability (degradation temperature (Td), and residue generated) of the PHB and PHBV standard samples. Samples of polymers (approximately 17 mg) were weighed and heated from 25 to 400 °C at a heating rate of 10 °C∙min^−1^, with a nitrogen gas flow of 50 mL∙min^−1^ [33]. The results were compared with the values obtained from the PHBV-STD.

#### 2.4.3. Mechanical Testing

##### PHA Film Formation

The PHA films were formed according to the “solvent molding” method of Gao et al. (2006) [34] with slight modifications. One gram of the biopolymer was dissolved in 100 mL of chloroform in a test tube, stirred vigorously (vortex) until a homogeneous solution was obtained, which was poured into a 20 cm diameter Petri dish. The chloroform was allowed to evaporate (inside an extraction hood) for 12 h on a level surface. The evaporation of the solvent resulted in the formation of films (0.1 ± 0.02 mm thick) on the petri dishes. The films were cut into 1 cm × 10 cm sheets, which were vacuum dried at 80 °C for 6 h. The films were kept at 50 ± 10% relative humidity for 7 days before the tensile tests.

##### Tensile Test

A tensile test based on the standard method of mechanical tensile properties for thin plastic sheets (ASTM D882-10) [35] was performed on a Brookfield CT3 texture analyzer (Middleboro, MA, USA) operated with an activation load of 0.044 N and a velocity of 0.5 mm∙s^−1^ [36] at room temperature (≈25 °C). Tensile strength, elongation at break, and Young’s modulus were determined through the stress–strain diagram obtained from the average of three samples. The results were compared with the values obtained from the PHBV-STD.

#### 2.4.4. Fourier Transform Infrared Spectroscopy with Attenuated Total Reflectance (FTIR-ATR) Analysis

FTIR-ATR analysis was performed to characterize the chemical structure of PHB, PHBV synthesized by BtE264, and PHBV-STD. The infrared spectra were obtained using a Spectrum GX System (Perkin-Elmer^®^, Springfield, IL, USA) equipped with an ATR accessory (Pike Technologies^®^, Madison, WI, USA). The analysis was performed in a spectral range of 4000–650 cm^−1^, with a resolution of 8 cm^−1^, with a total of 32 scans per sample [11,37].

#### 2.4.5. X-Ray Diffraction Analysis

X-ray diffraction (XRD) analysis was performed on a diffractometer (Rigaku Ultima IV^®^, Wilmington, MA, USA) equipped with a D/TeX detector and Cu-Ka radiation source in a range of 5–90° with a step size of 0.02° [38].

#### 2.4.6. Nuclear Magnetic Resonance (NMR) Analysis

^1^H NMR spectroscopy was used to determine the chemical structure of PHA polymers. In summary, approximately 20 mg of PHA sample was dissolved in deuterated chloroform, and ^1^H NMR spectra were recorded at 25 °C in a spectrometer [39].

## 3. Results and Discussion

### 3.1. BtE264 Growth and Substrate–Co-Substrate Consumption in BMP, BMP + LA, BMP + VA, and BMP + SPr

**Figure 1:**
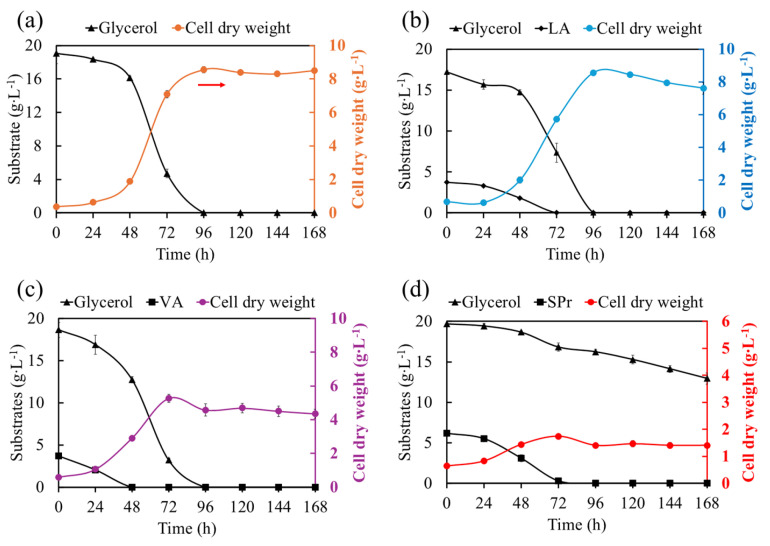
Growth kinetics and substrate consumption of BtE26: (**a**) BPM, (**b**) MBP + LA, (**c**) BPM + VA, and (**d**) BPM + SPr.

Figure 1 shows the evaluation of the effect of different co-substrates (AL, AV, and SPr) on the growth of BtE264 and its assimilation capacity as a source of co-carbon. Figure 1a shows the microorganism growth and glycerol consumption in the BPM; in the medium with glycerol as the sole carbon source, a growth value of 8.6 ± 0.19 g·L^−1^ at 96 h was reached, and the highest rate of glycerol consumption of 0.48 g∙L^−1^∙h^−1^ was observed from 48 to 72 h of culture. Figure 1b shows the growth, substrate, and co-substrate consumption profile in an BPM + LA; the addition of LA to the BPM resulted in a maximal growth of 8.6 ± 0.08 g·L^−1^ (96 h) at a lower glycerol consumption rate (0.31 g∙L^−1^∙h^−1^, from 48 to 72 h). This is related to the presence of LA in the culture media, which was fully consumed at a rate of 0.07 g∙L^−1^∙h^−1^, reflecting the simultaneous consumption of the two carbon sources [40]. Figure 1c and Figure 1d show the cell growth in BPM + VA and BPM + SPr, respectively.

Unlike in the BPM and BPM + LA, the highest microbial growth was recorded at 72 h culture, and lower biomass concentrations of 5.3 ± 0.24 g·L^−1^ and 1.7 ± 0.08 g·L^−1^ were obtained for BPM + VA and BPM + SPr, respectively. In the case of BPM + AV, the highest rate of glycerol and VA consumption was 0.40 g∙L^−1^∙h^−1^ (48–72 h) and 0.09 g∙L^−1^∙h^−1^ (24–48 h), respectively. On the other hand, glycerol consumption in the BPM + SPr treatment is limited from the beginning, with a 34% decrease at the end of the culture. Glycerol and SPr consumption rates between 48–72 h were 0.08 g∙L^−1^∙h^−1^ and 0.12 g∙L^−1^∙h^−1^, respectively.

The use of co-substrates in PHBV synthesis can be toxic in bacterial cultures, generating inhibition in cell growth, which depends on the assimilation metabolic capacity of the microorganism, and the co-substrate concentration used [18]. Ashby et al. (2018) [25] observed a decrease in the growth of *Burkholderia sacchari* DSM 17165 when the concentration of LA was increased from 0 to 2 g·L^−1^. Loo and Sudesh (2007) [41] reported that the inhibitory effects of 3HV precursors usually decrease in the order of SPr > VA > valerate salt. In this study, the addition of LA at 4 g·L^−1^ did not show an apparent inhibitory effect compared to that obtained in the BPM, representing an advantage of the use of LA for the PHBV production without inhibitory impact of the co-substrate on microbial growth. In contrast, the addition of VA and SPr to the BtE264 culture decreased the microbial growth by 48% and 84%, respectively, validating that although these co-substrates have an inhibitory effect on microbial growth, they are suitable for increasing the 3HV fraction on PHBV [22,42].

### 3.2. Production of PHBV by BtE264 in BPM, BPM + LA, BPM + VA, and BPM + SPr Culture Media

**Figure 2:**
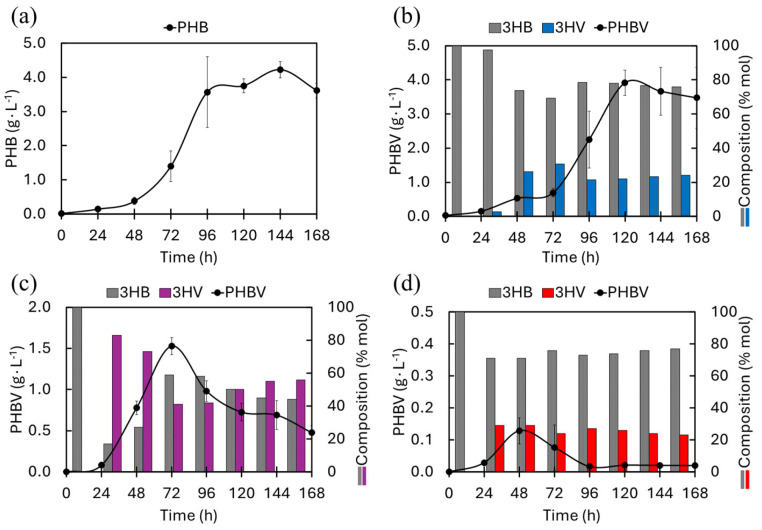
PHBV production by BtE264: (**a**) BPM, (**b**) BPM + LA, (**c**) BPM + VA, and (**d**) BPM + SPr.

Figure 2a shows the PHB production profile obtained in BPM, it is observed that the presence of glycerol as the only source of carbon leads to the production of 4.22 ± 0.24 g∙L^−1^ of PHB homopolymer at 144 h, with a dry biomass (Y_P/X_) product yield of 53% (Table 1). Figure 2b displays the production profile of PHBV and the molar percentages of 3HB and 3HV in the BPM + LA, where a production of 3.91 ± 0.37 g·L^−1^ (47% Y_P/X_) at 120 h with 22% mol of 3HV is recorded indicating that LA is the co-substrate leading to the highest production of PHBV by BtE264. Figure 2c,d depict the production profiles and molar percentages of 3HB and 3HV in BPM + VA and BPM + SPr media; lower PHBV production was obtained compared with MBP and BPM + LA media. In this case, the higher PHBV production in the BPM + VA medium was 1.53 ± 0.10 g·L^−1^ at 72 h (33% Y_P/X_) with 41% mol of 3HV, while in the BPM + SPr, a PHBV production was obtained with 0.13 ± 0.04 g L^−1^ at 48 h (13% Y_P/X_, 29% mol of 3HV), this being the lowest value compared with the other media.

Co-substrates are precursors added as an alternative or complementary carbon source, combined with the main substrate. These promote the synthesis of specific monomeric units by bacteria when limiting conditions are present in their environment [43,44]. On the other hand, the absence of a precursor for synthesizing 3HV hinders the microorganism’s formation of the PHBV co-polymer. This was observed in the BPM where BtE264 only synthesized the homopolymer PHB (Figure 1a). Although PHBV was not produced in the BPM, the production values obtained (4.22 ± 0.24 g∙L^−1^, 53% Y_P/X_) are higher than those reported by Blunt et al. (2023) [45] (Table 1). On the other hand, LA has been reported to be a viable co-substrate in the synthesis of 3HV [46], it has also been shown to be an inhibitor for PHA-producing bacteria when concentrations exceed 2 g∙L^−1^ [25]. In a similar study with *Ralstonia eutropha* (now *Cupriavidus necator*) for PHBV production using glucose and LA, Wang et al. (2013) [47] reported peak production values of 3.18 ± 0.25 g·L^−1^, with 21% mol of 3HV (72% Y_P/X_), which resembles its production values and % mol of 3HV with those obtained in this study by BtE264 in MBP + LA. Given that *R. eutropha* is considered to be the “model microorganism” in the synthesis of PHAs [48], comparing the results obtained by BtE264 provides us with a solid frame of reference, especially considering that, as far as is known, this study would be the first to report the production of PHBV by BtE264.

As previously reported, AV and SPr are among the most commonly used co-substrates as precursors in the synthesis of 3HV units, facilitating the incorporation of 3HV into PHBV polymer chains [49,50,51]. In this study, BtE264 in MBP + VA synthesized 83% mol of 3HV in the 24 h and achieved a PHBV production of 1.53 ± 0.10 g L^−1^ with 41% mol 3HV at 72 h. These results are lower than those reported by Urtuvia et al. (2020) [52] with the bacterium *Azotobacter vinelandii* (Table 1); however, it is worth mentioning that in the analysis by Urtuvia et al. (2020) [52], VA was added at 18 h of culture, which influenced cell growth and consequently PHBV production, suggesting that if the addition of VA in the MBP + VA medium was carried out at specific times (higher than the initial time), the inhibitory effect could be decreased by maximizing PHBV production by BtE264 [53]. SPr was the co-substrate that exerted the greatest inhibitory effect on BtE264; this is mostly attributed to a toxic effect on bacterial growth, accentuating as the added concentration increases [43,54]. Table 1 summarizes the PHA yields, 3HV composition, and biomass obtained from the most significant studies in the literature and compares them with the results of this research.

**Table 1:** PHBV biosynthesis by different bacterial strains and the effect of substrate and co-substrate.

Bacterial Strain	Substrate	Time (h)	Cell Dry Weight (g·L^−1^)	PHA Production (g·L^−1^)	3HV (mol %)	PHA Volumetric Productivity (g·L^−1^∙h^−1^)	Product Yield Y_P/X_ (gP∙g^−1^X)	Reference
BtE264	Glycerol	336	4.85 ± 0.15	2.51 ± 0.18	N/A	0.007 ± 0.0006	0.52 ± 0.04	[45]
*Ralstonia eutropha*	Glucose + LA	72	4.39 ± 0.44	3.18 ± 0.25	21	0.04 ± 0.003	0.72	[47]
*Burkholderia Sacchari*	Xylose + LA	72	3.3	1.5	43	0.02	0.45	[25]
*Azotobacter vinelandii*	Sucrose + AV	64	4.5 ± 0.8	2.8 ± 0.7	27	0.04 ± 0.01	0.61 ± 0.05	[52]
*Ralstonia eutropha*	SPr	96	0.87 ± 0.03	0.55 ± 0.05	92	0.005 ± 0.005	0.63 ± 0.07	[22]
BtE264	Glycerol	144	8.4 ± 0.16 ^A^	4.22 ± 0.24 ^A^	N/A	0.03 ± 0.002 ^A^	0.53 ± 0.04 ^A^	This study *
BtE264	Glycerol + LA	120	8.4 ± 0.04 ^A^	3.91 ± 0.37 ^A^	22	0.03 ± 0.003 ^A^	0.47 ± 0.03 ^A^	This study *
BtE2 64	Glycerol + VA	72	5.3 ± 0.24 ^B^	1.53 ± 0.10 ^B^	41	0.02 ± 0.001 ^B^	0.33 ± 0.01 ^B^	This study *
BtE264	Glycerol + SPr	48	1.0 ± 0.002 ^C^	0.13 ± 0.04 ^C^	29	0.003 ± 0.0008 ^C^	0.16 ± 0.05 ^C^	This study *

* The values in this study are the averages of three experimental units ± SD. Different letters in the same column indicate significant differences between treatments at α = 0.05.

### 3.3. Characterization of Thermal Properties by DSC and TGA

**Figure 3:**
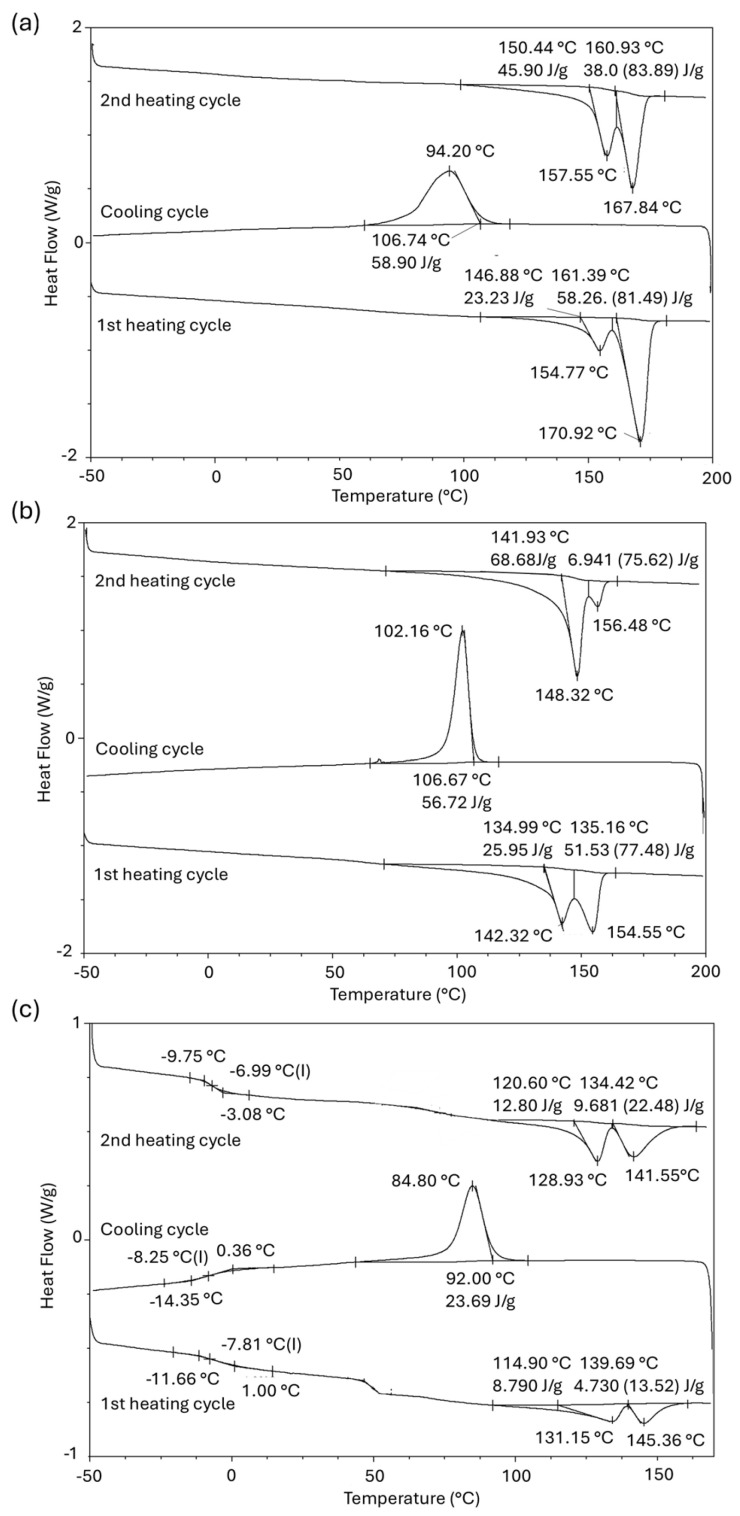
DSC thermograms of (**a**) PHB, (**b**) PHBV-STD, and (**c**) PHBV submitted to two heating–cooling cycles.

**Figure 4:**
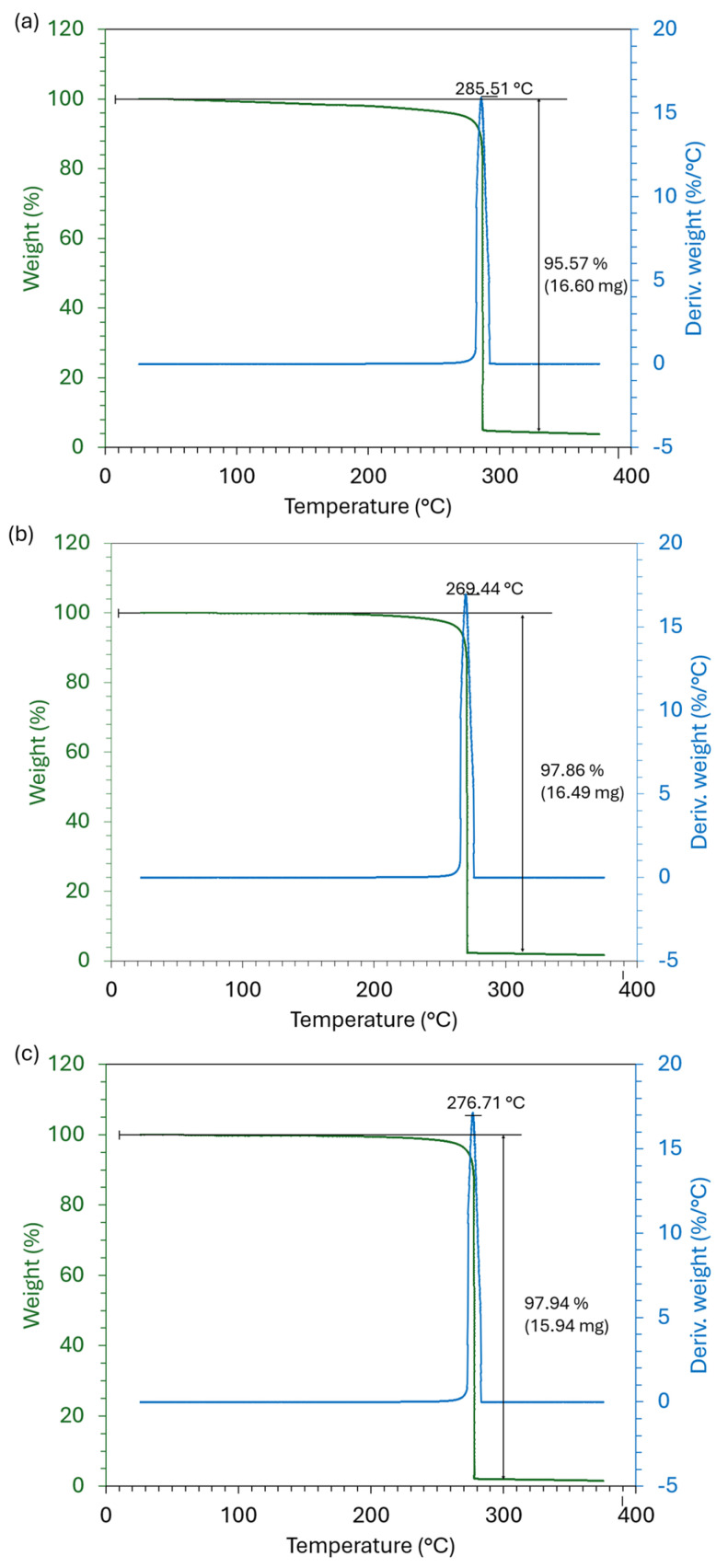
TGA thermograms of (**a**) PHB, (**b**) PHBV-STD, and (**c**) PHBV.

The thermal properties of PHA films are summarized in Figure 3 and Figure 4 and Table 2. The second heating cycle was used for the determination of thermal properties. The PHB and PHBV samples were analyzed by DSC and TGA, and the results were compared with a standard of PHBV (PHBV-STD). The PHB sample presented two fusion endotherms at 157 and 168 °C (∆Hm of 46 and 38 J·g^−1^). Also, it showed one exothermic crystallization peak at 94 °C (∆Hc of 59 J·g^−1^) in the cooling down run, and its Td was set at 285 °C based on the TGA thermogram. Similarly, the PHBV-STD sample showed two endothermic melting peaks at 148 and 156 °C (∆Hm of 69 and 7 J·g^−1^), the corresponding exothermic crystallization peak at 102 °C (∆Hc of 57 J·g^−1^) in the cooling down run, and the Td was 269 °C in the TGA thermogram. In contrast, the PHBV sample presented a Tg at −7 °C, in addition to the two endothermic fusion peaks at 129 and 141 °C (∆Hm of 13 and 10 J·g^−1^), the corresponding exothermal crystallization at 85 °C (∆Hc of 24 J·g^−1^), and the Td of 276 °C.

Semicrystalline polymers are made up of highly ordered zones (crystalline regions) and disordered zones (amorphous regions), which are responsible for the formation of compact and repetitive structures or undefined spatial arrangements in polymer chains [31]. Unlike the PHB and PHBV-STD samples, the PHBV sample presented a Tg value, which is attributed to a lower crystallinity in the PHBV sample (Table 2). The % of crystallinity was 39% and 35%, respectively, for the PHB and PHBV-STD samples. On the contrary, the PHBV samples showed a crystallinity of 21%. This translates into a more abundant and less restricted amorphous phase that triggered a more evident Tg in the DSC analysis [55]. On the other hand, for all the biopolymers analyzed, double melting peaks were observed, which is explained by the presence of two types of crystals that melt at different temperatures or recrystallization during melting as observed by Pracella et al. (2021) [56]. Semi-crystalline materials such as PHB tend to form regions with imperfect crystals that, when heated, melt and then reorganize into new, more ordered crystalline structures. These crystals are more stable and require more energy to melt, which produces the second fusion endotherm at a higher temperature [57].

In TGA analysis, samples followed a decomposition mechanism in a single thermic event involving an abrupt and well-defined drop in mass over a relatively narrow temperature range. For the PHB, PHBV-STD, and PHBV samples, the initial mass loss was 95, 98, and 98% at temperatures of 285, 269, and 276 °C, respectively. These results are similar to those reported by Urtuvia et al. (2023) [58] for PHBV (33% mol 3HV) produced by *Azotobacter vinelandii* OP.

**Table 2:** Reported thermal properties of PHB, PHBV-STD, and PHBV films.

		DSC				TGA				
Microorganism	PHA	T_g_ (°C)	T_m_ (°C)	∆H_m_ (J·g^−1^)	T_c_ (°C)	∆H_c_ (J·g^−1^)	Xc %	T_d_ (°C)	Weight Lost (%)	Reference
BtE264	PHB	−1.5	79.8	166.4	48.5	42.4	55	279	N/A	[59]
BtE264	PHB	−7.9	170	N/A	N/A	N/A	N/A	294	N/A	[45]
Goodfellow	PHB	N/A	163–169	76.9	111.7	62.9	52	N/A	N/A	[56]
*Methylocystis* sp.	PHBV (25% mol 3HV)	−4.8	163.9	41.9	67.6	N/A	38	N/A	N/A	[55]
*Burkholderia sacchari*	PHBV (88% mol 3HV)	−14	99.7	43.7	52.4	-42	40	N/A	N/A	[25]
*Azotobacter vinelandii* OP	PHBV (33% mol 3HV)	0.64	166	68.46	52.8	70.6	63	295	96.07	[58]
BtE264	PHB	N/A	157–168	46–38	94	59	57	285	95.57	This study
Sigma-Aldrich	PHBV-STD (8% mol 3HV)	N/A	148–156	69–7	102	57	69	269	97.86	This study
BtE264	PHBV (24% mol 3HV)	−7	129–141	13–10	85	24	21	276	97.94	This study

N/A = Not available.

### 3.4. Stress–Strain Diagram of PHB, PHBV-STD, and PHBV Films

The values of Young’s modulus, tensile strength, and film break elongation of PHB, PHBV-STD, and PHBV are presented in Table 3. The PHB presented the most rigid behavior, reflecting a high Young’s modulus, due to its stereochemical regularity, which implies that its atoms and functional groups are organized in space in a specific way, promoting the creation of crystalline zones, which contributed to greater rigidity between the polymer chains [59]. The presence of 3HV units in the polymer chains of PHBV decreases their stereochemical regularity due to the presence of an ethyl group that facilitates the degree of rotation and steric interactions with other groups in the molecule [59,60], which was observed with the decrease of Young’s modulus in the PHBV-STD sample compared to PHB. In the case of PHBV, a different behavior is observed, because the material manages to deform with less stress than PHB and PHB-STD, exhibiting a lower Young’s modulus and a higher percentage of elongation at break compared to the other samples. This is attributed to the increase in the mol fraction of 3HV in the PHBV sample, where a higher % mol of 3HV increases the amorphous phase in the polymeric structure decreasing the ordering of the chains, reflecting the importance of increasing the 3HV content in PHBV to improve flexibility and reduce the fragility of the biopolymer [11]. In this study, the value of break elongation obtained (3.1%) is similar to what is reported as suitable for film formation [58].

**Figure 5:**
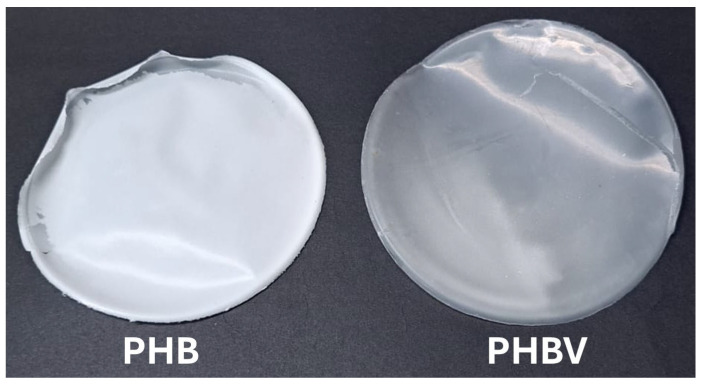
PHB and PHBV films.

Figure 5 shows the films formed of PHB and PHBV by solvent molding, which presented a uniform surface, without impurities and with good structural integrity, used for sheet forming for stress–strain tests.

### 3.5. Analysis of Fourier Transform Infrared Spectroscopy with Attenuated Total Reflectance (FTIR-ATR)

**Figure 6:**
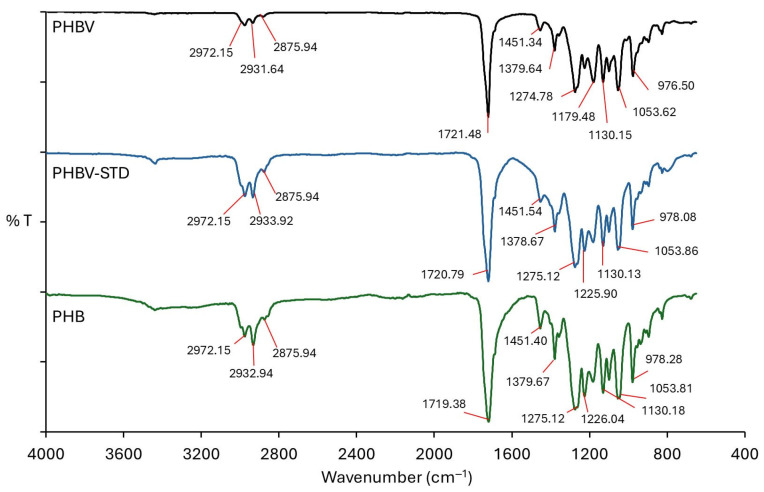
FTIR-ATR spectra of PHB, PHBV-STD, and PHBV.

Figure 6 shows the FTIR-ATR spectra of PHB, PHBV-STD, and PHBV. Characteristic bands of PHAs were identified, with an intense signal at 1720 cm^−1^ corresponding to the stretching of the carbonyl group (C=O) of the crystalline state of PHB and PHBV. The FTIR peak in the region 2933–2972 cm^−1^ is due to single-link C-H vibration [37]. The bands detected around 1451 and 1378 cm^−1^ were attributed to the asymmetric-symmetrical C-H stretch–flexion vibrations of methyl (CH_3_) and methylene (CH_2_) groups. Absorption bands were observed at 1275 and 1226 cm^−1^, corresponding to symmetrical stretch vibrations of group C-O-C, in addition to asymmetric tension vibrations C-O-C at 1060, 1099, and 1179 cm^−1^ [11,14]. The vibrations at 1275 and 1226 cm^−1^ were related to the crystalline region of the samples, observing a higher intensity in PHB versus PHBV, which is consistent with the analysis performed by DSC. It was observed that the PHBV produced by BtE264 had the same characteristic bands as the PHBV-STD, and agrees with that reported by other authors [11,61,62,63].

### 3.6. Diffraction Analysis in PHB, PHBV-STD, and PHBV Samples

**Figure 7:**
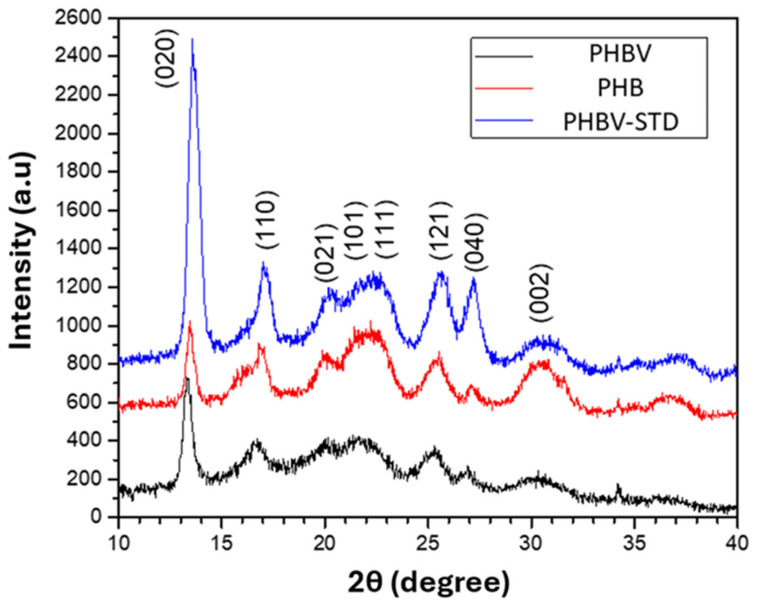
X-ray diffractograms of PHB, PHBV-STD, and PHBV samples.

Figure 7 shows the diffraction profile standard of the homopolymer, co-polymer, and commercial standard. At values of 2θ 13.60 the main diffraction peak appears being attributed to the plane (0 2 0) of the orthorhombic crystal structure type α of the 3HB) and 3HV units, the peaks that shows at 17.3°, 22.5°, 26.8°, and 29.8° correspond, respectively, to the diffraction planes (1 1 0), (1 0 1), (1 1 1), (1 2 1), (1 2 1), and (0 0 2), at values of 2 θ. The secondary peaks that appear at two θ of 17.30 correspond to the diffraction plane (1 1 0) of the crystalline latex of 3HB and 3HV [64]. The percentage of crystallinity values for PHB, PHBV-STD, and PHBV were 72.7, 73.3, and 5.8%, respectively, showing a similar trend to the DSC values (Table 2).

### 3.7. Nuclear Magnetic Resonance (^1^H NMR) Analysis of PHB, PHBV-STD, and PHBV Samples

**Figure 8:**
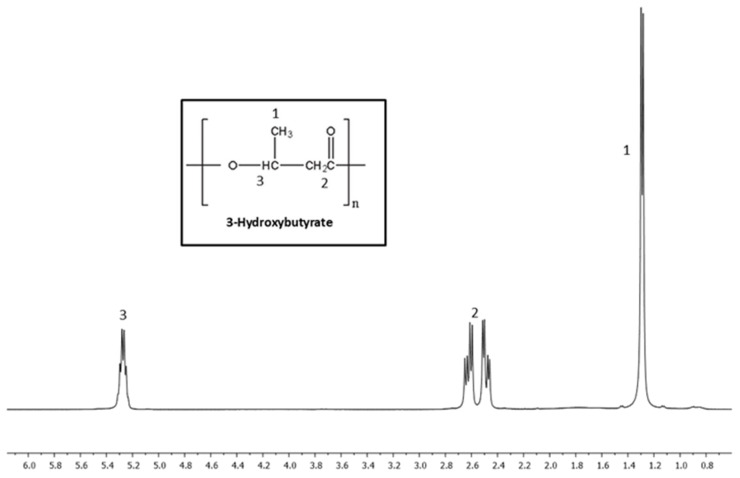
^1^H Nuclear Magnetic Resonance spectrum of PHB.

**Figure 9:**
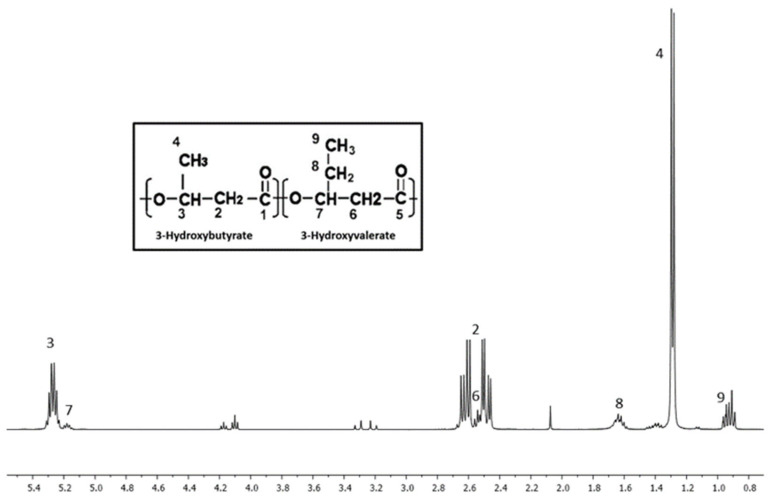
^1^H Nuclear Magnetic Resonance spectrum of PHBV-STD.

**Figure 10:**
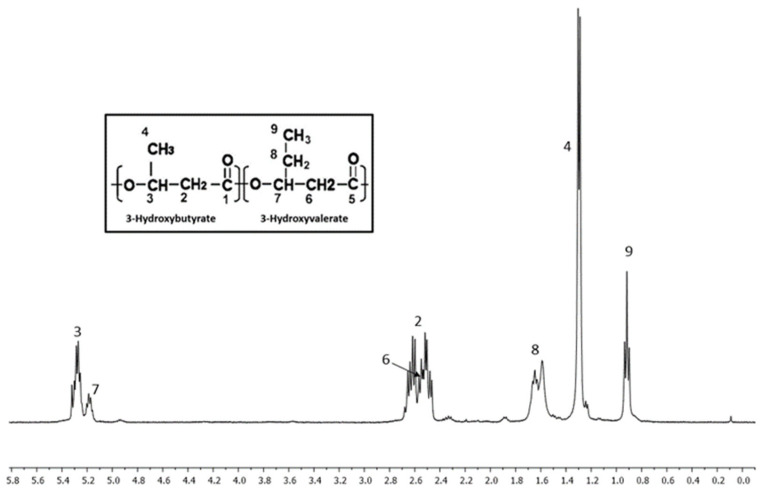
^1^H Nuclear Magnetic Resonance spectrum of PHBV.

Figure 8, Figure 9 and Figure 10 show NMR spectra for PHB, PHBV-STD, and PHBV samples. The ^1^H NMR spectrum of PHB (Figure 8) exhibited three different signals at 1.3, 2.5, and 5.3 ppm corresponding to the methyl terminal group (–CH_3_), to the methylene group (–CH_2_) adjacent to the carbonyl group (–CH_2_–CO–), and a methine group (–CH) bound to oxygen (–CHO–), which represent characteristic groups for PHB confirming their corresponding chemical structure [65]. Figure 9 and Figure 10 show the spectra of PHBV-STD and PHBV, where typically characteristic signals of the 3HB monomer were observed around 1.3, 2.5, and 5.3 ppm and 1.3, 2.4, and 5.3 for PHBV-STD and PHBV. Also, characteristic peaks of corresponding 3HV monomers 0.9 (–CH_3_), and 1.6 (–CH_2_) were observed, in addition to signals at 2.5 ppm of CH_2_ near the carbonyl group, as well as another one at 5.3 ppm of the methine group (–CH–) bound to oxygen, respectively [66], corroborating the structural identification of the PHBV co-polymer. In the case of PHBV-STD, additional signals of about 0.85 and 3.2 ppm attributed to solvent impurities (acetone, ethanol) were observed in the sample. These signals were not present in PHB and PHBV, indicating a higher purity in the samples synthesized by BtE264.

## 4. Conclusions

This study firstly reports the capability of *Burkholderia thailandensis* E264 (BtE264) to produce poly(3-hydroxybutyrate-co-3-hydroxyvalerate), with the simultaneous transformation of glycerol and levulinic acid. The synthesized PHBV displayed similar thermal properties (melting and degradation temperatures) to those reported by different bacterial strains. The use of levulinic acid as co-substrate influences the mechanical properties of the films, while the increase in % mol of 3HV led to a less rigid and more elastic material compared to PHBV-STD. The NMR indicates that BtE264 allows to produce 3HB and 3HV polymers in adequate structure conformation to form functional bioplastics.

Therefore, this work offers valuable insights into the production of sustainable bioplastics through the use of environmentally friendly technologies. It also contributes to the valorization of crude glycerol and lignocellulosic biomass as potential substrates for PHBV synthesis, emphasizing the necessity for further research aimed at optimizing and scaling the process to develop cost-effective and sustainable solutions tailored to practical requirements.

## Figures and Tables

**Table 3:** Tensile properties of PHB, PHBV-STD, and PHBV films *.

Origin	PHA	Young’s Module (MPa)	Tensile Strength (MPa)	Break Elongation (%)
BtE264	PHB	1194 ± 42 ^A^	10 ± 3.1 ^A^	0.9 ± 0.1 ^B^
Sigma-Aldrich	PHBV-STD (8% mol 3HV)	359 ± 48 ^B^	3.5 ± 1.2 ^B^	1.1 ± 0.1 ^B^
BtE264	PHBV (24% mol 3HV)	160 ± 47 ^B^	3.1 ± 0.9 ^B^	3.1 ± 0.7 ^A^

* The values are the average of three experimental units ± SD. Different letters in the same column mean significant differences between treatments at α = 0.05.

## Data Availability

The original contributions presented in this study are included in the article. Further inquiries can be directed to the corresponding author.

## Abbreviations

The following abbreviations are used in this manuscript:

BtE264	*Burkholderia thailandensis* E264
% Xc	percentage of crystallization
∆Hc	crystallization enthalpy
∆Hm	melting enthalpy
3HB	3-hydroxy butyrate
3HV	3-hydroxy valerate
ATCC	American Type Culture Collection
BPM	base production medium
BPM + LA	base production medium + levulinic acid
BPM + SPr	base production medium + sodium propionate
BPM + VA	base production medium + valeric acid
DSC	differential scanning calorimeter
EF	Erlenmeyer flask
FTIR-ATR	Fourier Transform Infrared Spectroscopy with Attenuated Total Reflectance
GC	gas chromatography
HPLC	high-performance liquid chromatography
LA	levulinic acid
NMR	Nuclear Magnetic Resonance
PDA	photodiode array detector
PHAs	polyhydroxyalkanoates
PHB	polyhydroxybutyrate
PHBV	poly (3-hydroxybutyrate-co-3-hydroxyvalerate)
PHBV-STD	poly (3-hydroxybutyrate-co-3-hydroxyvalerate)-Standard
RID	refractive index detector
SPr	sodium propionate
Tc	crystallization temperature
Td	degradation temperature
Tg	glass transition temperature
TGA	thermogravimetric analyzer
Tm	melting temperature
VA	valeric acid
XRD	X-ray diffraction
ΔH^0^m	calculated melting enthalpy
